# Towards ethical guidelines for e-health: JMIR Theme Issue on eHealth Ethics

**DOI:** 10.2196/jmir.2.1.e7

**Published:** 2000-03-31

**Authors:** Gunther Eysenbach

Note added after publication: For debate see also Letter to the Editor JMIR 2000; 2:e13 and the response

The Internet is a vast resource for consumers, but to realize its full potential it is necessary to ensure the quality of information, or at least to help consumers to assess the quality of information. While the Internet and interactive health communication clearly has the potential to make patient-physician encounters more effective [1], a recent paper published in the Journal of Medical Internet Research showed that only 19% of Scottish GPs felt that they were " to use the time more effectively" if patients come with Internet printouts [2].

The principal dilemma of the Internet is that its anarchic nature is desirable as it fosters open debate without censorship, but at the same time it raises quality problems that could inhibit its potential [3]. However, a single or centralized review process, institution, or agency to ensure quality is neither desirable or realistic, since the Internet is a decentralized, global medium: "Web 'publishers' of all stripes...should be free to post whatever they like and live with the consequences" [4]. We can call the resulting dilemma " farmer's dilemma," as any farmer battling with pests and weeds faces a similar problem: The more pesticides he uses, the more he inhibits the healthy growth of useful plants. Likewise, any "top-down" regulation on the Internet is prone to fail or to destroy "healthy" communication [3].

Instead, quality management of health information on the Internet depends on "bottom-up" mechanisms and essentially rests on four pillars - the " E's" (see Figure 1):

- Educating consumers,
- Encouraging self-regulation of health information providers,
- Evaluating information by third parties, and
- Enforcement, in case of fraudulent or positively harmful information.

**Figure 1:**
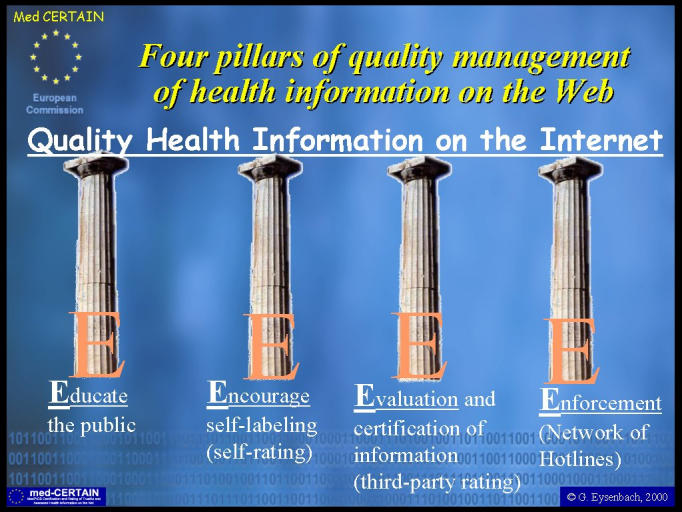
The four pillars of quality health information

## Educating consumers

First, consumers need to be educated on how to "filter" [3] information, i.e. how to discriminate trustworthy information from less trustworthy information. Some basic criteria consumers could look at include authorship, sources of information, potential bias, and date of publication [4]. Other groups have developed interactive Internet tools which help users to evaluate information, i.e. to assess the quality themselves (http://www.quick.org.uk, http://www.discern.org.uk, http://hitiweb.mitretek.org/iq/default.asp) [5]. For example, the DISCERN instrument [6] is a tool for assessing the quality of written patient information material. It has shown to be reliable for printed health information, but its validity has not been established for electronic information. An Internet version is currently being developed and awaits evaluation.

## Encouragement of self-regulation and self-rating

The second pillar consists of two different components. One component is self-rating of information providers, i.e. publishing of metainformation, which allows users to locate and filter information automatically [3]. Information providers could, for example, include metainformation which indicates the target group of the information [7].

**Figure 2:**
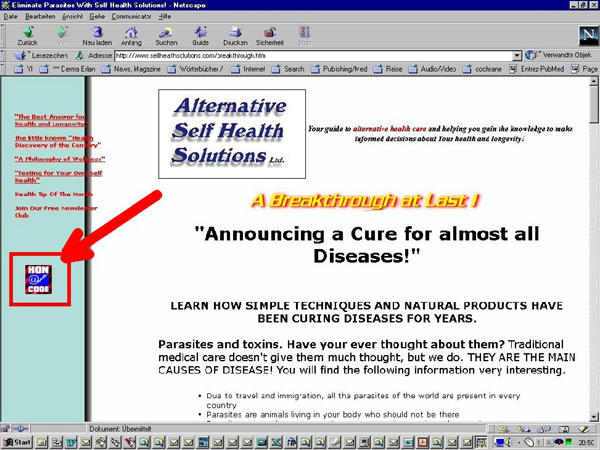
Fraudulent health information providers can mislead consumers by simply self-publishing logos that suggest trustworthy health information

Another component is self-regulation. The Health on the Net Foundation has been among the first to suggest an ethical code for web publishers [8]. However, the suggested self-publishing of a logo (the HON-Logo) on the website of the information provider is problematic, perhaps even counterproductive; even quackery sites proudly display the logo (see Figure 2), and many consumers (and even health professionals) misunderstand the HON-logo as an "award". As the Health on the Net Foundation says, the HON-Logo was a "marketing trick," to make the HON Code well known.

However, without third-party evaluation and enforcement (which both will be addressed by the medCERTAIN project, see below), this ethical code is a toothless tiger. A more sophisticated system is needed, for example where the logo or "seal of approval" is generated dynamically by a third party (as planned in the medCERTAIN project described below).

Self-regulation of industry suddenly became a hot topic in September 1999, when one of the leading health portals, http://DrKoop.com (see Figure 3a), was criticised for a lack of "web ethics." In an article published in the New York Times (see Figure 3b), the site (partly owned by former U.S. surgeon general Everett Koop) was critized for having an inadequate distinction between editorial content and promotion. For example, DrKoop.com published a list of hospitals designated as "the most innovative across the country," not revealing that these hospitals actually paid for the listing. Moreover, the site was criticized for calling advertisers "partners". Additionally, it was said that DrKoop.com violated medical ethics (the guidelines of the American Medical Assocation) by making money referring patients to other physicians: on the website, DrKoop.com published listings of clinical trials, receiving a fee paid by the clinical research company (Quintiles) for each patient "referral" - without revealing this fact.

**Figure 3a:**
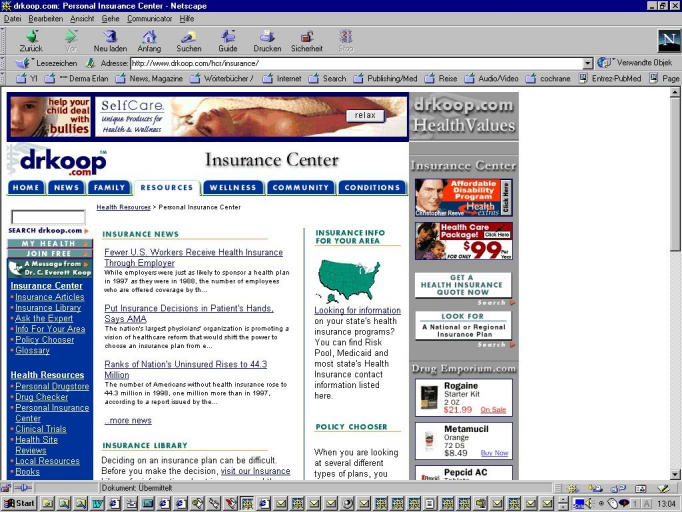
The DrKoop.com website with blurred borders between editorial content and advertising..

**Figure 3b:**
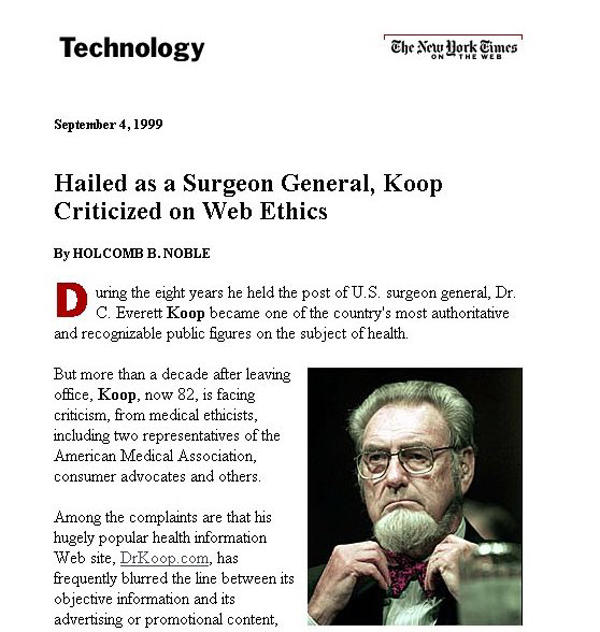
...was reproached by the New York Times due to their lack of " ethics."

**Figure 4:**
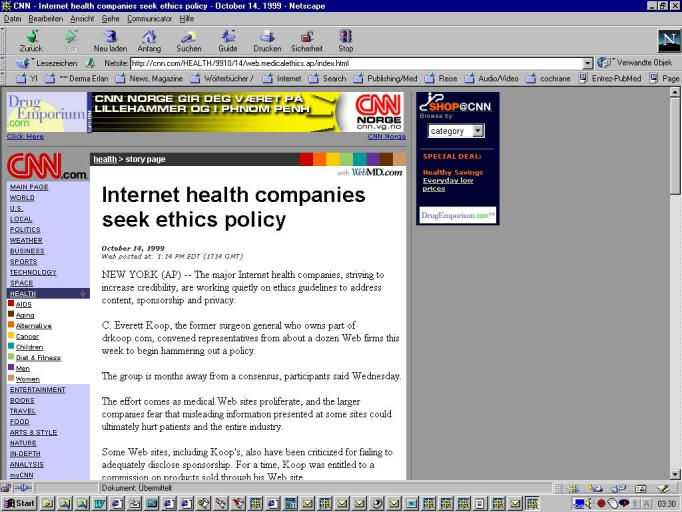
CNN coverage of DrKoop.com's ethics policy summit

The case was taken up by other media, and the loss of trust and reputation for DrKoop.com was considerable. As a consequence of this, DrKoop.com convened representatives from about a dozen Web firms in October 1999 to begin hammering out an ethics policy (see Figure 4). However, the meeting ended with few concrete commitments.

A few days later, on Wednesday, October 13, 1999, George Lundberg, editor in chief of the health portal Medscape and former editor of the Journal of the American Medical Association (fired for publishing a survey on sexual attitudes that coincided with President Clinton's impeachment), gave a keynote speech at the annual meeting of the Internet Healthcare Coalition (IHC), a non-profit organization mainly consisting of companies in the eHealth business [9], where he said: "The essence of professionalism is self-governance. Just as the International Committee of Medical Journal Editors, founded in 1978, has set the standards for how medical journal authors and editors should behave, the leaders of the e-health information enterprise should now set common standards for ethical behavior."

Two days later, the IHC announced it would organize a working summit in Washington D.C., on January 31st - February 1st, 2000, to forge a set of ethical principles for health-related Web sites. The summit was chaired by Helga Rippen and Ahmad Risk, both IHC Board Members (and also editorial board members of the Journal of Medical Internet Research). About fifty international experts were invited to the summit to meet in the rooms of the WHO/PAHO (World Health Organization/Pan-American Health Organization) in Washington. The attendees developed the guiding principles for a eHealth Ethics Code which addresses guidelines for Internet health information providers around issues like quality of content; commercial behaviour; privacy, security and confidentiality; and use of the Internet in the practice of health care. A draft of this code is published in this issue of JMIR [10].

## Evaluation

As mentioned above, self-regulation is only one step towards quality on the Web; two other steps are third-party evaluation and enforcement.

A recently-launched project named medCERTAIN (MedPICS Certification and Rating of Trustworthy Health Information on the Net, http://www.medcertain.org/), funded by the European Union under the "Action Plan on promoting safer use of the Internet by combating illegal and harmful content on global networks" will tackle these issues, and therefore build directly on the work performed by the IHC. The project follows up the idea that the quality of health information and interactive applications on the Internet cannot and should not be controlled by a central body or authority, but instead information and applications must be evaluated and "labeled" in a decentralised and distributed way [3,11,12]. Labeling means to provide metainformation, i.e. to provide information about information, which may be descriptive or evaluative [7]. These information labels may be attached to other information on the web, and displayed whenever a user accesses a website. The medCERTAIN consortium plans to use the PICS standard (Platform for Internet Content Selection), which is compatible with every modern web browser. Whenever a user accesses a fraudulent web site, the browser requests a label from a third party database, and can for example display a warning. Within the medCERTAIN project, a technical infrastructure is currently being developed which allows individuals, organizations, associations, societies, and other entities to digitally label (rate, evaluate, peer-review, give quality seals to...) online published health information using labels consisting of a standard computer-readable vocabulary (metainformation). The medCERTAIN consortium will also create different levels of certification for publishers of health information on the web (ranging from simple quality seals indicating the "good standing" of the site to "gold" quality seals indicating that the site has been peer-reviewed externally).

As mentioned above, the medCERTAIN project is one project funded under the "Action Plan on promoting safer use of the Internet by combating illegal and harmful content on global networks," adopted on December 21, 1998, by the Council of the European Union: "This action plan is a European Commission proposal for a number of initiatives from 1 January 1999 to 31 December 2002 with a total budget of 25 million Euro. The initiatives, created in close co-operation with industry, Member States and users, include a network of hot-lines, support for self-regulation, developing technical measures and awareness initiatives. The aim of the Action Plan is to ensure implementation of the various initiatives on how to deal with undesirable content on the Internet. It is designed to support non-regulatory initiatives for promoting safer use of the Internet" (http://www2.echo.lu/iap/).

While most of the initiatives under the Internet Action Plan are targeting content which could be harmful for children (pornography, violence), medCERTAIN proposes a system to establish a certification and rating system for rating and filtering of health information.

## Enforcement

Enforcement requires feedback channels for worried consumers, procedures for evaluating complaints, and the possibility of appropriate measures such as labeling (blacklisting) of information providers who, for example, seriously violate ethical or legal standards. The EU Action Plan contains the concept of hotlines allowing concerned consumers to channel concerns; the medCERTAIN project will also contain feedback channels for consumers, which may lead to the re-evaluation of a site and retraction of a rating/certification.

## Further articles in this issue

Aside from the draft version of the Washington Code of eHealth Ethics [10], this issue of JMIR further contains two reviews tackling the difficult issues of practicing medicine on the Web without a pre-existing patient-physician relationship (e.g. responding to unsolicited patient emails) [13] and the chances and challenges of e-psychotherapy [14]. Moreover we look into problems which are related to traditional problems of publishing ethics and academic misconduct [15,16]. Interestingly, this report on cyberplagiarism and the activities of the Journal of Medical Internet Research in this field, including a new policy that every submitted manuscript will be electronically scanned for plagiarism, have already attracted some media coverage [17] and may stimulate thought and debate in the publishing world about informatics tools which may detect academic misconduct and thereby enforce ethical conduct in publishing and research.

Gunther Eysenbach, MD

Editor,

Journal of Medical Internet Research
